# Advanced Cutaneous Leiomyosarcoma of the Forearm

**DOI:** 10.3390/dermatopathology8010008

**Published:** 2021-02-27

**Authors:** Gerardo Cazzato, Maria Chiara Sergi, Sara Sablone, Anna Colagrande, Teresa Lettini, Francesco Fanelli, Umberto Orsini, Giuseppe Ingravallo

**Affiliations:** 1 Section of Pathology, Department of Emergency and Organ Transplantation, University of Bari Aldo Moro, 70124 Bari, Italy; gerycazzato@hotmail.it (G.C.); anna.colagrande@gmail.com (A.C.); francescofanelli@gmail.com (F.F.); giuseppe.ingravallo@uniba.it (G.I.); 2 Section of Medical Oncology, Department of Biomedical Sciences and Clinical Oncology (DIMO), University of Bari Aldo Moro, 70124 Bari, Italy; sergimariachiara@gmail.com; 3 Section of Forensic Medicine, Department of Interdisciplinary Medicine, University of Bari Aldo Moro, 70124 Bari, Italy; sarasabloneml@gmail.com; 4 Orthopedics Section, Department of Medical Sciences of Basis, Neurosciences and Organs of Sense, University of Bari Aldo Moro, 70124 Bari, Italy; umberto.orsini@policlinico.ba.it

**Keywords:** primary cutaneous leiomyosarcoma (PCL), skin, differential diagnosis

## Abstract

Leiomyosarcoma is a malignant smooth muscle neoplasm, which is traditionally divided into superficial and deep tumors. Superficial leiomyosarcomas are quite rare entities, accounting for approximately 7% of soft tissue neoplasms and 0.04% of all cancers. Here we describe a rare case of advanced primary cutaneous leiomyosarcoma (PCL) in a 93-year-old woman, highlighting the considerable size of the lesion and the correct surgical and oncological management. The clinical story began about 4 years ago, and the neoplasia was treated only with local radiotherapy, but the patient suffered from a dramatic volumetric increase of the right arm sarcoma one year ago. Then, an amputation of the limb was performed without following adjuvant chemotherapy. Currently, she does not show signs of recurrence and is in good shape.

## 1. Introduction

Leiomyosarcoma is a malignant smooth muscle neoplasm, which is traditionally divided into superficial and deep tumors. Superficial leiomyosarcomas are quite rare entities, accounting for approximately 7% of soft tissue neoplasms and 0.04% of all cancers [1]. Based on localization, superficial tumors are further subclassified in dermal and subcutaneous forms; traditionally, it is supposed that the dermal forms have a less aggressive behavior than lesions primitively localized in the subcutaneous [1]. We report a rare case of primary cutaneous leiomyosarcoma (PCL) highlighting the considerable size of the lesion and the correct surgical and oncological management. About 3 years after the initial diagnosis, the neoplastic lesion reported an almost “dramatic” volumetric increase in the previous 6 months.

## 2. Case Report

**Figure 1:**
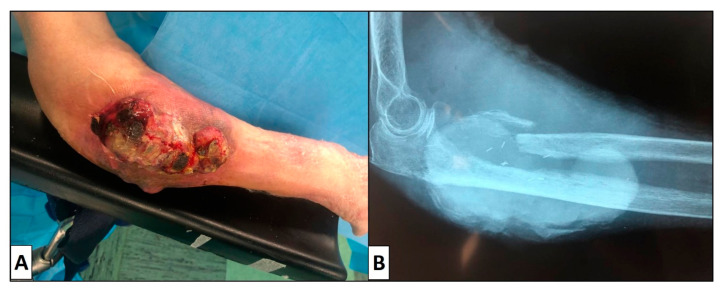
Ulcerated cutaneous lesion (**A**). Radiographic image (**B**) shows a big lesion of soft tissue that has infiltrated underlying bone plans, in particular proximal radio, which is almost completely eroded. The proximal ulna also appears infiltrated and partly rare.

A 93-year-old woman presented herself to the Complex Operating Unit of Orthopedics of Aldo Moro University of Bari for a worsening of her health conditions. About 3 years earlier, he had been diagnosed with a primary cutaneous leiomyosarcoma at another hospital, which had been treated with local radiotherapy. In recent months, he had noticed a progressive increase in the size of the lesion. On clinical examination, the woman showed a lesion of 10.3 × 9.1 × 2.4 cm located in the right arm, extensively ulcerated, with polycyclic contours, with hemorrhagic zone (Figure 1A). Radiographic image confirmed the destructive appearance of the neoplasm, which, in addition to infiltrating the subcutis and the underlying bone (humerus) (Figure 1B).

After an interdisciplinary consultation, it was decided to amputate the limb as the patient’s age did not indicate the use of systemic chemotherapy, a much-debated point in the Literature (see Section 3). Pathological gross examination described a surgical sample consisting of a lozenge of skin, subcutis, and muscle fascia of 11.2 × 9.0 × 3.0 cm, that was almost entirely occupied by an ulcerated, grey-whitish lesion with irregular contours, which extensively infiltrated the subcutis, with no apparent cleavage plane. Adequate sampling of the lesion was carried out, paying particular attention to the surgical resection margins.

**Figure 2:**
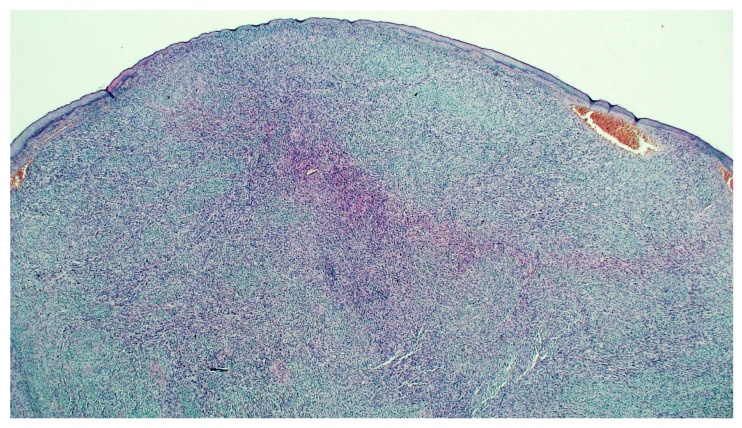
Histopathological features of the primary cutaneous leiomiosarcoma at low magnification.

**Figure 3:**
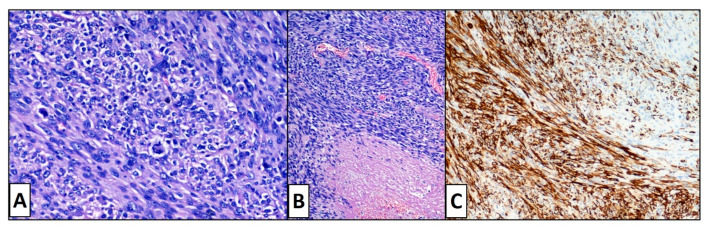
Histopathological features of the leiomyosarcoma. At high magnification the neoplastic spindle cells showed pleomorphic and hyperchromatic nuclei with atypical mitotic figure and extensive necrosis (**A**,**B**). The tumor cells displayed consistent smooth muscle actin immunoreactivity (**C**).

After tissue processing, paraffin embedding, microtome cutting and routine haematoxylin-eosin staining, the microscopic examination showed at low magnification (Figure 2), at the dermal level, the presence of a proliferation of variously intertwined atypical spindle cells was described, which tended to ulcerate the overlying epidermis and infiltrate the subcutaneous. Immunohistochemistry was positive for muscle markers such as smooth muscle actin (SMA). (Figure 3C). About 1 year after the amputation surgery, the patient showed no signs of recurrence and is in good health. Furthermore, the decision made in the multidisciplinary team not to submit the patient to adjuvant chemotherapy after surgery, by virtue of her age, appears to have had no negative effects. Additionally, it was not considered necessary to subject the patient to radiotherapy, as the minimum criteria of oncological surgical radicality were met (see Section 3).

At higher magnification, the neoplastic cells showed pleomorphic, nucleolated, hyperchromatic nuclei, and moderate to abundant eosinophilic cytoplasm: numerous typical and atypical mitotic figures, extensive necrosis, and hemorrhages were present together with areas of apparent dedifferentiation (Figure 3A,B). The tumor cells displayed consistent smooth muscle actin (Figure 3C), desmin immunoreactivity, and sporadic positivity for Actin HHF35. Based on the above features, the diagnosis of cutaneous leiomyosarcoma was made.

## 3. Discussion

Cutaneous leiomyosarcoma is a rare soft tissue sarcoma that has traditionally been interpreted to originate in the normal pilo-erector muscles of the dermis [2]. It is a rare entity mostly described in the population after 65 years of age [3] and with a male/female ratio of 3:1 or 4:1 according to Bonamonte et al. [4]. Our case is particularly interesting for two main reasons: the patient’s clinical history and the bad outcome. In fact, a first diagnosis of PCL was made 3 years before and after biopsy, a local radiotherapy treatment was opted for. All this had not resulted in a cure of the disease, but only in an attempt to control the progression.

In fact, about 3 years after the initial diagnosis, the patient reported an almost “dramatic” volumetric increase in the previous 6 months with a general decline in clinical conditions. In the Literature [5], it is considered that PCL tends to have no necrosis and marked nuclear pleomorphism: in our case, however, both of these characteristics were clearly present, suggesting that the tumor had undergone a more aggressive biological and histological evolution. A total-body CT was conducted to rule out the possibility that it was a metastatic lesion.

Clinically, PCL can be misinterpreted as a keloid, granulomatous lesion (such as cutaneous tuberculosis or other typical or atypical mycobacteriosis, deep skin fungal infection, skin sarcoidosis) or as a benign neoplastic lesion (epidermoid cyst, dermatofibroma, lipoma, fibroma, leiomyoma, neurofibroma) or malignant (basal and squamous cell carcinoma, melanoma, Kaposi’s sarcoma, and other soft tissue tumors, especially dermatofibrosarcoma protuberans). From the histological point of view, PCL must be differentiated from fibrosarcoma, melanoma, malignant tumor of the peripheral nerve sheath, synovial sarcoma, malignant fibrous histiocytoma, pseudosarcomatous squamous cell carcinoma, and dermatofibrosarcoma protuberans [4]. Therefore, it is crucial to emphasize that immunohistochemistry (i.e., smooth muscle actin, desmin, vimentin, cytokeratins, and S-100 protein immunoreactivity) is of fundamental aid to build a correct diagnosis. As a rule, the expression of smooth muscle actin, desmin, and vimentin by tumor cells and negativity for the remaining markers allows for differentiation from the aforementioned neoplasms.

Standard treatment is surgical resection with free lateral margins of 3–5 cm including subcutaneous tissue reaching the fascia [6]. Kraft and Fletcher identified margin status as the most important predictor of recurrence: in cutaneous LMS (leiomyosarcoma), which is usually an indolent pathology, the involvement of subcutaneous or extension into the fat, leads to a worse prognosis and local recurrence of 40%. According to the analysis of 36 cases of PCL (primary cutaneous leiomyosarcoma) conducted by Massi et al. the relapse-free interval is 22–84 months from diagnosis [6]. To reduce the risk of cancer recurrence, patients with large lesions (>5 cm), tumor-positive excision margins, high-grade LMS (G2, G3), and local relapse receive radiotherapy. The regimen provides high energetic photons (6–18 MV) within 6 weeks after surgery with a cumulative dose of 40–50 Gray [3].

Adjuvant chemotherapy is a controversial issue. Administration of doxorubicin and ifosfamide is the most widely used therapy, showing an increased overall survival (OS) and odds ratio (OR) for local recurrence of 0.73 (95% CI 0.56–0.94; *p* = 0.02) [7].

In the metastatic setting, polychemotherapy includes doxorubicin plus ifosfamide or doxorubicin plus dacarbazine, and after progression with an anthracycline, trabectedin is the common second-line therapy [8].

Alternative treatment can be pegylated liposomal doxorubicin or epirubicin. Gemcitabine plus docetaxel or gemcitabine alone achieve disappointing results [9]. Orally multikinase inhibitors and ICI are studied in soft tissue sarcomas.

Sorafenib and regorafenib showed no clinical benefit in LMS [7], whereas in the PALETTE trial Pazopanib (a multitargeted tyrosine kinase inhibitor with activity against VEGF 1-2-3 and PDGF) was studied at dose levels of 800 mg/die vs. placebo after the failure of standard chemotherapy. In the LMS subgroup, median OS was of 16.7 months and PFS was 20.1 weeks for pazopanib and respectively 8.1 and 14.1 for placebo. Therefore, FDA has approved the use of Pazopanib for the treatment of patients with advanced soft tissue sarcoma who have received prior chemotherapy [8].

Combination blockade with nivolumab plus ipilimumab demonstrates efficacy with a response rate of 18% and a median OS of 14.3 months. Nevertheless, the data are scarce, because of the rarity of this cancer [10]. Hence, systemic therapy in PCL remains anectodal and a choice of an individual institution’s tumor board.
